# Serum Vascular Adhesion Protein-1 and Endothelial Dysfunction in Hepatic Cirrhosis: Searching for New Prognostic Markers

**DOI:** 10.3390/ijms25137309

**Published:** 2024-07-03

**Authors:** Silvano Fasolato, Emanuela Bonaiuto, Monica Rossetto, Paola Vanzani, Fabio Ceccato, Fabio Vittadello, Lucio Zennaro, Adelio Rigo, Enzo Mammano, Paolo Angeli, Patrizia Pontisso, Maria Luisa Di Paolo

**Affiliations:** 1 Department of Medicine, Padua University Hospital, 35128 Padua, Italy; 2 Department of Molecular Medicine, University of Padua, 35128 Padua, Italymonica.rossetto@unipd.it (M.R.); lucio.zennaro@unipd.it (L.Z.); marialuisa.dipaolo@unipd.it (M.L.D.P.); 3 Unit of Surgical Oncology of the Esophagus and Digestive Tract, Veneto Institute of Oncology IOV-IRCCS, 35128 Padua, Italyenzo.mammano@aopd.veneto.it (E.M.); 4 Explora s.n.c.—Research and Statistical Analysis, 35010 Padua, Italy; 5 Nazionale di Biostrutture e Biosistemi (INBB), Consorzio Interuniversitario Istituto, Viale Medaglie d’Oro, 00136 Roma, Italy; 6 Department of Medicine, Medical Clinic 5, University Hospital of Padua, 35128 Padua, Italy; pangeli@unipd.it (P.A.); patrizia@unipd.it (P.P.)

**Keywords:** vascular adhesion protein-1, alcoholic cirrhosis, hepatocellular carcinoma, adhesion molecules, endothelial dysfunction

## Abstract

Endothelial dysfunction plays a key role in the development of liver cirrhosis. Among the biomarkers of endothelial dysfunction, the soluble form of Vascular Adhesion Protein-1 (sVAP-1) is an unconventional and less known adhesion molecule endowed also with amine oxidase activity. The aim of this study was to explore and correlate the behavior of sVAP-1 with that of the soluble vascular cell adhesion molecule-1 (sVCAM-1) and intercellular adhesion molecule-1 (sICAM-1) and with the severity of liver cirrhosis. A cross-sectional study was carried out by enrolling 28 controls, 59 cirrhotic patients without hepatocellular carcinoma, and 56 patients with hepatocellular carcinoma (HCC), mainly caused by alcohol abuse. The levels of adhesion molecules and of the pro-inflammatory cytokines (IL-6 and TNF-αα) were determined by immunoassay and the enzymatic activity of sVAP-1 by a fluorometric assay. In non-diabetic patients without HCC, a specific behavior of sVAP-1 was highlighted. Differently from sVCAM-1, sICAM-1, and cytokines, the sVAP-1 level was significantly increased only in the early stage of disease, and then, it decreased in the last stage (866 ± 390 ng/mL vs. 545 ± 316 ng/mL, in Child–Pugh class A vs. C, respectively, *p* < 0.05). Bivariate analysis correlates sVAP-1 to sVCAM-1, in the absence of HCC (Spearman’s rho = 0.403, *p* < 0.01). Multiple linear regression analysis revealed that sVCAM-1 appears to be a predictor of sVAP-1 (β coefficient = 0.374, *p* = 0.021). In conclusion, in non-diabetic and non-HCC cirrhotic patients, sVAP-1 may be a potential prognostic biomarker that, together with sVCAM-1 and pro-inflammatory cytokines, may provide information on the progression of sinusoidal liver endothelium damage.

## 1. Introduction

Liver diseases usually occur in response to chronic hepatocellular injury caused by various etiologies, such as alcohol abuse, viral infections, bile duct damage, and non-alcoholic fatty liver disease. Hepatocellular carcinoma (HCC), the fourth leading cause of cancer-related death in the world, is mainly diagnosed in patients with underlying chronic liver disease [1]. Progression from chronic liver disease to cirrhosis and to HCC is driven by persistent inflammation, fibrosis, and aberrant hepatocyte regeneration [1,2,3,4]. In such a process, liver endothelial dysfunction plays a crucial role, with possible prognostic implications [5]. Indeed, the liver sinusoidal endothelial cells (LSECs) display unique structural and phenotypic features and have a critical role in immune response during liver injury [6]. In particular, data in the literature [4,6,7,8,9] support a key role of the hepatic tissue microenvironment in liver inflammatory diseases, where adhesion molecules (intercellular adhesion molecule (ICAM-1), vascular cell adhesion molecule-1 (VCAM-1) and vascular adhesion protein-1 (VAP-1), chemokines and cytokines (interleukins and tumor necrosis factor alpha (TNF-α)), could be involved in a possible mechanism for neutrophil-mediated liver damage.

Among the adhesion molecules, while VCAM-1 and ICAM-1 and E-selectin are well- known markers of endothelial activation and dysfunction [10,11] VAP-1 is less known. VAP-1 has the particularity to be both an adhesion molecule and an enzyme, a semicarbazide-sensitive amine oxidase (SSAO). As an enzyme, it catalyzes the oxidative deamination of primary amines to produce the corresponding aldehyde, ammonium, and hydrogen peroxide, a reactive oxygen species that is involved in oxidative stress [12]. VAP-1 is constitutively expressed at high levels in adipocytes, in smooth muscle cells, on the surface of liver sinusoidal endothelial cells [13], and in non-inflamed vascular endothelial cells, where it is stored within intracellular vesicles. During inflammation, VAP-1 expression is induced in endothelial cells and it is then translocated from cytosolic vesicles to the luminal surface of blood vessels. Here, as an ectoenzyme, VAP-1 can function physically as an adhesion receptor and, at the same time, through its catalytic activity it can regulate the recruitment of cells/leukocytes [8,12,14]. Thus, VAP-1 seems to be involved both in transient covalent binding of leukocytes and in the autocrine and paracrine regulation of other molecules participating in the inflammation process. Indeed, VAP-1 activity induces the expression of chemokines, other adhesion proteins (such as ICAM-1, VCAM-1, and E-selectin), and the activation of transcription factors, such as NF-κB, mainly by its reaction product H_2_O_2_, which is involved in various signaling pathways [12,14]. This dualistic function is needed for rolling and transmigrating leukocytes through the endothelium [15]. The cleavage product by matrix metalloproteinases of the membrane-bound VAP-1, the so-called “circulating or soluble” VAP-1 (sVAP-1), can be found in the sera of healthy human subjects and its level is increased in some inflammatory diseases [12], like inflammatory liver diseases [16,17], diabetes [18], systemic sclerosis [19], and Alzheimer ’s disease [20]. The soluble/serum form of VAP-1 (sVAP-1) that derives from the hepatic endothelial bed represents the major source of the soluble and enzymatically active form of VAP-1 in liver diseases [16,21].

The influence of VAP-1 on leukocyte transmigration, inflammation, and oxidative stress [22] suggests that it could have a crucial role in the pathogenesis of diverse human diseases, making it an important diagnostic and prognostic biomarker [23]. In particular, sVAP-1 has been associated with a damaged endothelium, such as in kidney transplant recipients [24], and endothelium of cancer vasculature [25]. It should be noted that tumor endothelium is phenotypically different from normal endothelium; for example, it responds abnormally to inflammatory stimuli due to the suppressive effect exerted by angiogenic growth factors (such as VEGF), as already shown in the expression of adhesion molecules ICAM-1 and VCAM-1 escaping immune surveillance [26].

In some tumors, the sVAP-1 level has been shown to have a predictive meaning, and its modulation has been found to be associated with disease progression. Although the literature data are sometimes conflicting, several studies have shown that low sVAP-1 levels are associated with a worse prognosis in colorectal [27,28], gastric [29,30], and thyroid cancer [31]. Also, a direct correlation of tissue VAP-1 expression has been found with progression of tumor invasion and metastasis in breast carcinoma [32], while a reduced expression of VAP-1 was found in prostate cancer [33].

Little is known about the behavior of sVAP-1 in the progression of liver disease, even if several studies documented an important role for sVAP-1 as a diagnostic and prognostic factor in chronic liver diseases (alcoholic liver disease, primary biliary cirrhosis, and primary sclerosing cholangitis) [16,21]. High levels of sVAP-1 have been detected in patients with non-alcoholic fatty liver disease (NAFLD) [4], alcoholic liver cirrhosis, and primary human HCC compared to controls [21,34,35]; VAP-1 was also proposed as a pharmacological target in non-alcoholic steatohepatitis [34]. Few studies have analyzed the correlation of sVAP-1 levels to the severity of liver disease: an increase in sVAP-1 level with progressive fibrosis has been observed in chronic hepatitis C [36] and an increased expression of VAP-1 was found in tumor endothelium of HCC [37], while the inhibition of enzymatic activity of VAP-1 was found to suppress HCC growth in mice [38].

No studies have been carried out so far on the comparison between the behavior of sVAP-1 and that of other classical adhesion molecules and markers of endothelial dysfunction (sVCAM-1 and sICAM-1) in liver diseases.

To search for new prognostic markers of the progression of liver disease, the aim of this study was to investigate in alcoholic liver cirrhosis the relationships between sVAP-1 and the other well-known adhesion molecule markers of endothelial dysfunction (sVCAM-1 and sICAM-1) and the potential correlation of their serum level on the stages of severity of disease (Child–Pugh classification). With this purpose, the serum levels of adhesion molecules (sVAP-1, sICAM-1, and sVCAM-1) and of inflammatory cytokines (IL-6 and TNF-α) were measured and analyzed in patients with different extents of severity of alcoholic liver cirrhosis, in the presence or absence of HCC. Our data indicate that in non-diabetic patients without HCC, the levels of sVAP-1 were significantly increased only in the early stage of disease, and then, it decreased in the most severe stage of cirrhosis.

## 2. Results

### 2.1. Patient Characteristics

The main baseline demographics and clinical characteristics of the patients included in this study are reported in Table 1.

About half of the 115 cirrhotic patients had hepatocellular carcinoma (HCC-1). Alcohol was the main cause of cirrhosis in comparison to viral hepatitis (90 vs. 25 patients). As cirrhosis affects mainly the male gender, the majority of the recruited patients were male (83%), both in the HCC-0 and HCC-1 groups, and well matched for age. The healthy control group included men aged 22 to 52 years old, without any health problems, such as hypertension or cardiac dysfunction, which are very common over 60 years old. Females were not included in the control group, as previous studies reported a slight increase in sVAP-1 in females in comparison with males in young individuals (35–45 years old) [39]. Furthermore, no difference in sVAP-1 by gender in an older control group has also been reported (43–59 years old) [4].

Patients were selected based on the degree of the severity of the disease and according to the inclusion criteria reported in Section 4.1; at the end of enrolment 29% of patients were found to be affected by diabetes mellitus. After the measurements of the various biomarkers, diabetic patients were analyzed separately.

The severity of the cirrhosis was calculated according to the Child–Pugh classification (CHILD), which is based on the values of serum bilirubin, albumin, on the international normalized ratio (INR) blood test levels, and on the presence and gravity of ascites and encephalopathy. This classification allows patients to be grouped into three classes: class A (good hepatic function, Child–Pugh score: 5–6), B (moderately impaired hepatic function, Child–Pugh score: 7–9), and C (advanced hepatic dysfunction, Child–Pugh score: 10–15). Considering the Child–Pugh classes (A, B, and C), patients without HCC (HCC-0) were homogeneously distributed, while patients with HCC (HCC-1) mostly belonged to classes A and B. As expected, αFP was significantly higher in the group of patients with hepatocellular carcinoma HCC-1 (*p*< 0.05).

### 2.2. Markers of Endothelial Activation and Inflammation in the Presence and Absence of Hepatocellular Carcinoma

**Figure 1:**
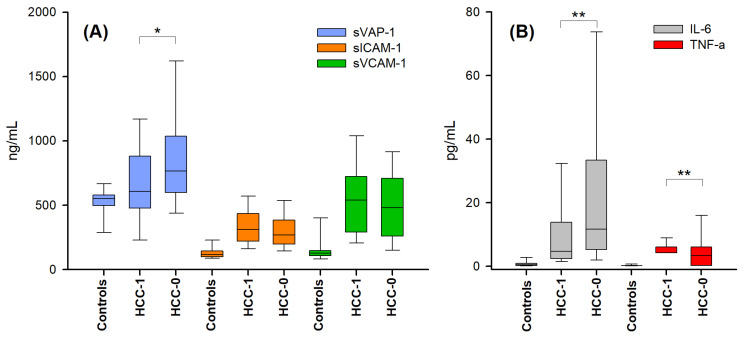
Adhesion molecules (**A**) and pro-inflammatory cytokines (**B**) in healthy controls, and cirrhotic patients with (HCC-1) and without (HCC-0) hepatocellular carcinoma. The level of sVAP-1 in the control group was lower only than that of the HCC-0 group, while the levels of IL-6, TNF-α, sVCAM-1, and sICAM-1 in the control group were significantly lower than those found in the HCC-0 or HCC-1 groups (Kruskal–Wallis test, *p* < 0.05, not marked in the figure). The serum levels of sVAP-1, IL-6, and TNF-α in the HCC-0 group were significantly different if compared to those of the HCC-1 group (Kruskal–Wallis test, Dunn’s multiple comparisons. *: *p* < 0.05, **: *p* < 0.01). The bottom and the top lines of box plots represent the first and third quartiles (25th percentile and the 75th percentile), respectively; the continuous lines mark the median values; and whiskers above and below the box indicate the 90th and 10th percentiles.

As a first step, serum levels of the various biomarkers (IL-6, TNF-α, sVCAM-1, sCAM-1, and sVAP-1) were assessed in the different groups of patients and in the controls. As shown in Figure 1, the concentrations of IL-6, TNF-α, sVCAM-1, and sICAM-1 were significantly lower in the control group than in the groups of patients with cirrhosis HCC-0 and HCC-1 (Kruskal–Wallis test, *p* < 0.05). Furthermore, significant differences were observed between the two groups of the patients (HCC-0 vs. HCC-1) in the case of sVAP-1, IL-6, and TNF-α (Kruskal–Wallis test, *p* < 0.05).

These data are congruent with a condition of endothelial activation and inflammation in the patient groups and in agreement with previous studies, which reported an increase in sVCAM-1, sICAM-1, and cytokines in cirrhotic patients in the absence of HCC (HCC-0) compared to healthy controls [10,40]. This behavior might be partially dependent also on the mean age of the control group, that is younger than those of the cirrhotic groups. Indeed, adhesion molecules and cytokines were found to increase with age due to various conditions of endothelial activation and inflammation caused by physiological aging processes [41].

Therefore, focusing on the two patient groups, by the comparison of cirrhotic patients with hepatocarcinama (HCC-1) and without hepatocarcinoma (HCC-0), significant differences were found only for the concentrations of cytokines (Figure 1B) and of sVAP-1 (Figure 1A) (HCC-0 vs. HCC-1: mean value 18.4 vs. 10.0 pg/mL for IL-6; mean value 3.6 vs. 5.3 pg/mL for TNF-α; *p* < 0.001; mean value 840 vs. 683 ng/mL for sVAP-1; *p* < 0.05). In addition, it was found that within the HCC-1 group, no significant differences emerged between the alcoholic and viral etiologies for all tested biomarkers (Table S1).

**Figure 2:**
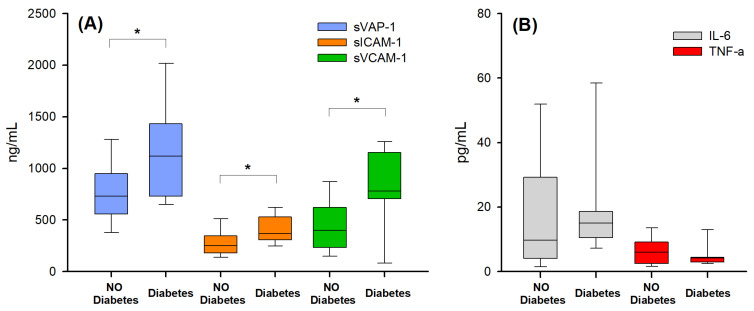
Serum levels of adhesion molecules (**A**) and pro-inflammatory cytokines (**B**) in cirrhotic patients without hepatocellular carcinoma (HCC-0), in presence and in absence of diabetes. A significant increase in the serum concentration of the three adhesion molecules were found in diabetic patients (panel (**A**)). * *p* < 0.05; Mann–Whitney *U* test. The bottom and the top lines of box plots represent the first and third quartiles (25th percentile and the 75th percentile), respectively; the continuous lines mark the median values; and whiskers above and below the box indicate the 90th and 10th percentiles.

Since sVAP-1 and other soluble adhesion molecules’ (VCAM-1, ICAM-1, and E-selectin) concentrations are known to increase in diabetic patients [18,42], and diabetes is a frequent comorbidity in cirrhosis, being diagnosed in 28% of our patients, its possible effect on the concentrations of the various biomarkers was analyzed. The effect of diabetes emerged only in the group of patients without hepatocellular carcinoma (HCC-0), where the levels of all the adhesion molecules analyzed were significantly higher in diabetic than in non-diabetic patients (Figure 2A), while no significant difference was found for inflammatory cytokines (Figure 2B). No significant difference between the diabetic and non-diabetic patients was found in the patients with HCC (HCC-1; Table S2).

On the basis of these results, in order to avoid any *bias* on the serum levels of adhesion molecules, only patients without diabetes were included in subsequent analysis. After the exclusion of diabetic patients, no significant difference emerged between patients with hepatocellular carcinoma (HCC-1) and without HCC (HCC-0) for all the tested biomarkers (Table 2, *p* > 0.05, Mann–Whitney *U* test).

### 2.3. Biomarker Correlation with Disease Severity

Previous studies in cirrhotic patients of various etiology reported an increase in the serum levels of sVCAM-1, sICAM-1, IL-6, and TNF-α in relation to the severity of the disease [10,40]. Additionally, sVCAM-1 levels were also found to increase in the severe fibrosis stages and hepatic inflammation [43].

With these premises, and on the basis of the effect of diabetes on adhesion molecules, the relationship between the levels of the above-reported biomarkers and of sVAP-1 with the severity of the cirrhosis (according to the Child–Pugh’s score: groups A, B, C), were analyzed in the sub-groups of non-diabetic patients, (HCC-0)’ and (HCC-1)’.

**Figure 3:**
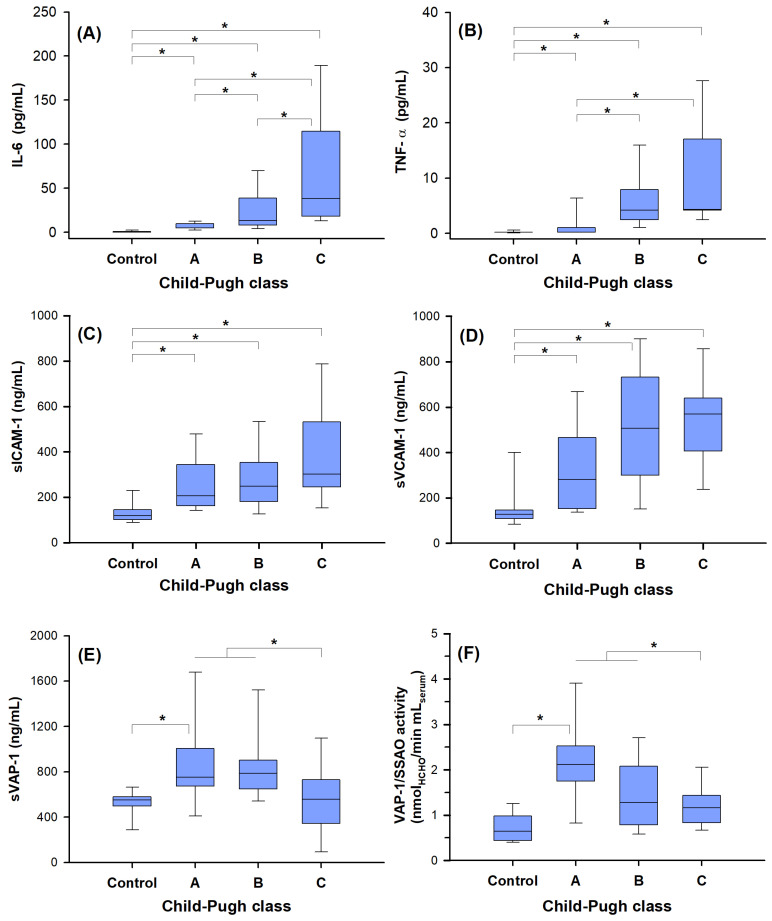
Serum concentration of the adhesion molecules and pro-inflammatory cytokines in non-diabetic cirrhotic patients without hepatocellular carcinoma (HCC-0)’ and in healthy volunteers (control). Classes of severity were formed according to Child–Pugh score (CP A/B/C). Panels (**A**,**B**) show the significant increase in the serum levels of IL-6 and TNF-α, respectively, with the severity of cirrhosis. IL-6: *p* < 0.05 for controls vs. CP A, B, or C; *p* < 0.05 for CP A vs. B or C, and for CP B vs. C; TNF-α: *p* < 0.05 of controls vs. CP B or C; *p* < 0.05 for CP A vs. B or C. Panels (**C**,**D**) show the trends in sICAM-1 and sVCAM-1, both of which increase with the severity of cirrhosis. For sICAM-1: *p* < 0.05 controls vs. for CP A, B, or C; *p* = 0.050 for CP A vs. C; for sVCAM-1: *p* < 0.05 for controls vs. CP A, B, or C; *p* < 0.05 for CP A vs. CP C. Panels (**E**,**F**) compare the sVAP-1 protein concentrations and the serum SSAO/VAP-1 activity. An increase in both the protein sVAP-1 and its activity (VAP-1/SSAO) was found by the comparison of healthy individuals with the CP A group (*p* < 0.05). By comparing the patients of the groups CP “A + B” vs. CP “C” a significant decrease was found for sVAP-1 (*p* = 0.013) and for SSAO/VAP-1 activity (*p* = 0.017). A significant decrease (*p* < 0.05) of SSAO/VAP-1 activity can be observed also for CP A vs. B or C (panel (**F**)). The bottom and the top lines of box plots represent the first and third quartiles (25th percentile and the 75th percentile), respectively; the continuous lines mark the median values; and whiskers above and below the box indicate the 90th and 10th percentiles (*): *p* < 0.05; Kruskal–Wallis test, Dunn’s multiple comparisons.

Interestingly, the presence of hepatocellular carcinoma influenced the results found for the patients with cirrhosis. In the group of patients with hepatocarcinoma (HCC-1)’, no significant difference emerged among the three Child–Pugh classes (CP A/B/C) for all the biomarkers (Table S3). Differently, in the patients without HCC (HCC-0)’ the serum levels of the cytokines (Figure 3A,B) increased in parallel with the CP class, while the levels of adhesion molecules sICAM-1 and sVCAM-1 (Figure 3C,D) were increased significantly only with respect to the control group but not to the CP class (*p* > 0.5; Kruskal–Wallis test, Dunn’s multiple comparisons).

Differently to sICAM-1 and sVCAM-1, it should be noted that sVAP-1 levels, after a marked increase in the early stage of disease (866 ± 390 ng/mL, ranked CP A), decreased in the last stage of disease (545 ± 316 ng/mL ranked CP C) (Figure 3E).

This specific behavior of the sVAP-1 protein, detected by immunoassay, corresponded to an equivalent trend determined by assaying its enzymatic activity. The results shown in Figure 3F clearly demonstrate how the SSAO/VAP-1 activity in plasma is strongly increased in the early stage of the disease with respect to control samples, and then, it decreases (CP A vs. CP C: 2.12 vs. 1.2 nmol _HCHO_ × min^−1^ × mL^−1^_serum_, Kruskal–Wallis nonparametric test, and Dunn’s multiple comparisons, *p* < 0.05) in agreement with data obtained by immunodetection of the sVAP-1 protein (Figure 3F).

### 2.4. Redox Biomarkers with Disease Severity in Cirrhotic Patients without HCC

Given the specific behavior of sVAP-1 compared to the other biomarkers in the absence of hepatocellular carcinoma and diabetes, the “redox” status of these patients was evaluated, by measuring two indirect oxidative stress markers: the carbonyl groups of the protein (an oxidative stress-induced modification of proteins), and the plasmatic level of nitrites and nitrates (produced when nitric oxide system is imbalanced).

**Figure 4:**
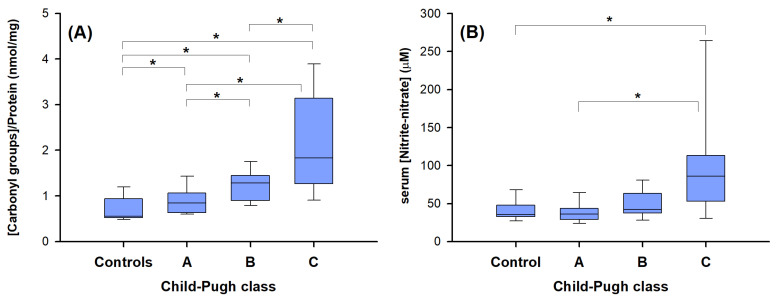
Biomarkers of oxidative damage in non-diabetic patients without hepatocellular carcinoma (HCC-0)’ along the Child–Pugh classes; carbonyl groups bound to plasmatic protein (**A**) and nitrite and nitrate concentration in serum (**B**). A significant increase in the carbonyl groups was found among the controls vs. the groups A, B, and C and among A, B, and C (panel A). The serum nitrite-nitrate concentration shows a significant increase in CP C vs. control or vs. CP A (*p* < 0.05). The bottom and the top lines of box plots represent the first and third quartiles (25th percentile and the 75th percentile), respectively; the continuous lines mark the median values; and whiskers above and below the box indicate the 90th and 10th percentiles. * *p* < 0.05; Kruskal–Wallis test, Dunn’s multiple comparisons.

The results in Figure 4 clearly show a significant increase in the serum levels of the two redox biomarkers in the last stage of cirrhosis (CP score “C”), supporting a condition of increased oxidative stress and damage [44].

### 2.5. Potential Correlations between the Various Biomarkers

As the above-reported results highlighted a different behavior of sVAP-1 compared to the other adhesion molecules (sVCAM-1, sICAM-1) and cytokines (TNF-α and IL-6), the potential correlations between all the various biomarkers were analyzed. The results of the bivariate analysis are reported in Table 3.

Among the adhesion molecules, sVCAM-1 and sICAM-1 are significantly correlated, both in the presence and absence of hepatocellular carcinoma. Differently, s-VAP-1 shows a correlation with sVCAM-1 only in the absence of hepatocellular carcinoma (Spearman’s rho = 0.403, *p* < 0.01). Also, TNF-α and IL-6 are correlated but only in patients without HCC.

Focusing on sVAP-1, the variables HCC, diabetes, TNF-α, IL-6, ICAM-1, and VCAM-1 were inserted in a multiple linear regression analysis, in order to evaluate a linear relationship with the serum level of sVAP-1.

The results reported in Table 4 show that only the mean levels of sVCAM-1 appear to be an independent predictor of sVAP-1. This result is in agreement with the above-reported correlation for these two biomolecules (Table 3) and support previous findings on the crucial involvement of sVAP-1 in the recruitment of CD16+ monocytes across liver sinusoidal endothelium, in combination with other molecules, such as sVCAM-1 [9,45].

## 3. Discussion and Conclusions

The present study has evaluated and compared the serum levels of sVAP-1 and two other adhesion molecules (sVCAM-1 and sICAM-1) in patients with alcoholic cirrhosis; in particular, the possible influence of the presence of underlying liver cancer and of the severity of the disease were analyzed. The levels of the pro-inflammatory cytokines IL-6 and TNFα, mediators classically associated with inflammation, were also determined in the same samples.

At first, the comparison between the patients in the absence and presence of HCC was performed. Among the adhesion molecules, only sVAP-1 was found to be significantly higher in patients without HCC in comparison to patients with HCC, and all the biomarkers were found at lower levels in the control group than in the patient groups, in agreement with other studies [4,10,21].

Previous studies reported that diabetes induces an increase in the serum level of cytokines of adhesion molecules (sCAM-1, sVCAM-1, and sVAP-1) [42,46,47]; therefore, in the second step of our analysis this variable was considered, because 29% of our patients presented diabetes, a common comorbidity of cirrhosis. The levels of all the adhesion molecules, but not of the cytokines, were found to be significantly increased in patients with diabetes and without HCC. This result is not surprising, because cirrhosis is often associated with an altered glucose metabolism and a condition of insulin resistance. During the progression of liver disease, there is an increase in insulin resistance. In advanced stages of cirrhosis, progressive histological and functional alterations such as increased insulin secretion as well as decreased hepatic insulin clearance (to compensate for peripheral insulin resistance) can be observed, along with rearrangement of hepatic cell types, change in insulin receptors expression, and distribution beyond change in the expression of glucose transporters (GLUTs). In the specific case of VAP-1, in sinusoidal endothelial cells of an experimental model of diabetes, an increase in VAP-1 and GLUT expression resulted in concomitant changes in insulin levels, which was deduced to represent an attempt to regulate blood glucose levels. In fact, VAP-1 activity has insulin-like activities, capable of stimulating glucose uptake and mitigating hyperglycemia, counterbalancing the effects of insulin resistance [47,48,49]. The hydrogen peroxide produced by VAP-1 activity also has the effect of promoting the condition of oxidative stress, and the expression of other adhesion molecules and pro-inflammatory cytokine (such as TNF-α). These events can also activate shedding and release of the soluble form of VAP-1 (probably through matrix metalloproteases [50]), which subsequently promotes oxidative damage as a secondary consequence of its ectopic amine oxidase activity. Thus, this dual function of VAP-1, adhesion molecule and amine oxidase, strongly differentiates its role and physio-pathological function in comparison to the other adhesion molecules.

The effect of diabetes on the adhesion molecules therefore highlights that this comorbidity of cirrhosis must be taken into account in future studies, in particular for sVAP-1, since the inclusion of diabetic patients may lead to misleading evaluations of their serum levels.

Consequently, when excluding patients with diabetes as a comorbidity from the analyses of non-diabetic patients the average levels of all the tested biomarkers in cirrhotic patients were found not to be significantly affected by the presence or absence of HCC. Conversely, Kemic et al. [35] reported an influence of HCC on the level of sVAP-1 in cirrhotic patients: they found serum levels of sVAP-1 to be higher in the presence of HCC than in the absence of HCC. Unfortunately, no information about diabetes comorbidity was reported.

Finally, in non-diabetic patients, a specific behavior of sVAP-1 in relation to the severity of liver disease (Child–Pugh score), was highlighted. In non-diabetic patients without hepatocellular carcinoma, sVAP-1 increased in the early stage, but then it decreased in the last stage of disease. Differently, the other adhesion molecules and the pro-inflammatory cytokines did not decrease with the severity of cirrhosis, as previously reported [10,40,51,52,53]. The high levels of cytokines, sVCAM-1, and sICAM-1 in patients in the advanced stage of cirrhosis correlate with the increased levels of the markers of oxidative stress (carbonyl groups of serum proteins and nitrites and nitrates plasmatic levels) and support a “picture” of increased inflammation, endothelial dysfunction, and oxidative stress with the progression of liver disease.

The novel and main finding of our study is the different behavior of sVAP-1, that, unlike the other investigated biomarkers, decreases with the severity of cirrhosis. Even if previous studies reported an increased level of sVAP-1 with progression of fibrosis in non-alcoholic fatty liver disease [4] and in chronic hepatitis C infection [36], our results do not conflict with these data, because such previous studies included patients with diabetes (about 44% of diabetic patients vs. 28% of controls in the cohort of Weston et al. [4]), which probably determined the increased sVAP-1 levels.

**Figure 5:**
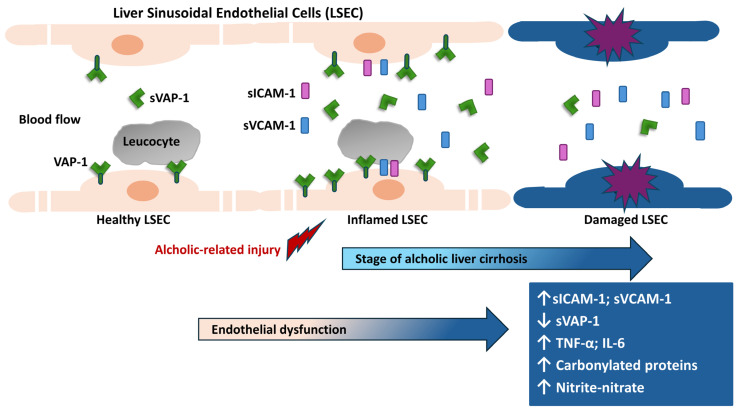
Effect of the progression of liver sinusoidal endothelial dysfunction and severity of liver cirrhosis linked to the level of serum VAP-1 (sVAP-1). The alcoholic-related injury induces a condition of liver sinusoidal endothelial dysfunction, with an increase in the serum level of adhesion molecules (sICAM-1, sVCAM-1, and sVAP-1) and cytokines (IL-6 and TNF-α). In the last and most severe stage of cirrhosis, under the condition of strong oxidative stress, LSEC are strongly damaged with a consequent decrease in the production and release of sVAP-1. Differently, sICAM-1, sVCAM-1, cytokines, carbonylated proteins, and nitrite-nitrate serum level are increased in comparison to the initial stage, being more systemic biomarkers.

The particular behavior of sVAP-1 in non-diabetic cirrhotic patients not affected by HCC, namely, its decrease with the severity, may be explained by the fact that in the last stage of cirrhosis there is an important damage of the liver sinusoidal endothelium. Thus, being the membrane-bound form of VAP-1 highly expressed by the human sinusoidal endothelial cells, with the progression of cirrhosis and the subsequent damage to the endothelium, the surface of functional liver sinusoidal endothelium is diminished, with a consequent decrease in the production and release of sVAP-1 (Figure 5). This conclusion is also supported by the fact that in the case of a massive hepatic necrosis, such as that induced by paracetamol, no increase in sVAP-1 level was reported [16].

Conversely, in the case of cirrhotic patients with HCC, the membrane-bound form of VAP-1 is also expressed by tumor vascular endothelium [37] and it might contribute to produce its serum form (sVAP-1), and its level consequently does not decrease with the progression of the disease. This last finding should be confirmed by further investigations performed on a larger sample size of patients with end-stage liver disease with HCC.

It is noteworthy that other studies reported a decreased level of sVAP-1 with the progression of disease for other types of cancer, such as in colorectal [27] and gastric cancers [28,29], where sVAP-1 was proposed as a valuable parameter for predicting poor prognosis and lymph node and hepatic metastasis. Since the ability to avoid immune attack is one of the hallmarks of cancer, VAP-1 might be a defensive factor against tumor progression, being involved in binding to the vasculature of cancer tissue of the tumor-infiltrating lymphocytes (TILs), which, presumably, can kill the malignant cells [25]. Therefore, the serum level of VAP-1 (sVAP-1) might reflect the local antitumor response in some type of cancers [25,37]. In contrast to this potential defensive role of VAP-1, the use of VAP-1/SSAO inhibitors in a murine model of HCC attenuated infiltration of myeloid-derived suppressor cells, tumor growth, and neo-angiogenesis in the tumor tissue [38]. These partly conflicting data suggest that the precise role of VAP-1 in the hepatic tumor microenvironment is not completely clear and requires further studies.

The other adhesion molecules, sVCAM-1 and sICAM-1, behaved instead as more systemic biomarkers of endothelial activation and dysfunction [10]. Since ICAM-1 and VCAM-1 are induced and expressed not only by vascular endothelial cells, but also by other types of cells, the production of their soluble forms remains high also in the last stage of cirrhosis, when sinusoidal endothelial cells are damaged. In particular, ICAM-1 is also expressed by lymphocytes, cancer-associated fibroblasts, and some neoplastic cells [54], while VCAM-1 is expressed also by macrophages, dendritic cells, and Kupfer cells [51]. It should also be noted that our statistical correlation analyses indicate that only sVCAM-1, known to be a potential biomarker in the last stage of fibrosis [43], was found to be a predictor of sVAP-1.

Our study acknowledges some limitations. The exclusion of patients with diabetes from the whole set of recruited patients reduced the sample size, in particular that of the patients affected by HCC at the last stage. Consequently, recruitment of other selected patients might be useful to verify our results in the HCC group and also the soluble E-selectin, another marker of endothelial dysfunction and linked to VAP-1 activity, might be interesting to include in future studies. The observational nature of our study does not allow us to exactly determine if and to what extent sVAP-1 is derived from the liver. To generalize our findings, the use of animal experiments and liver biopsy could enable us to demonstrate that, in the specific case of cirrhosis induced by abuse of alcohol, the liver is closely linked to the circulating sVAP-1 level.

In conclusion, this study demonstrates the different and specific behavior of sVAP-1 in comparison with the other two adhesion molecules as the severity of liver cirrhosis progresses, in the case of alcoholic liver disease. sVAP-1 seems to be a potential prognostic biomarker that, if monitored over time together with sVCAM-1 and pro-inflammatory cytokines, may provide information on the damage progression of the sinusoidal liver endothelium in non-diabetic patients with cirrhosis. This frequent comorbidity of cirrhosis should be considered in future studies on adhesion molecules. Future investigations are needed both to evaluate the effect of diabetes on sVAP-1 levels in patients with HCC and to identify and to validate the optimal cut-off values for a possible use of sVAP-1 in clinical practice.

## 4. Materials and Methods

### 4.1. Patient Selection and Plasma Collection

This cross-sectional study was conducted on 142 participants, aged between 22 and 89 years old. The participants were categorized into three main categories: heathy controls (*n* = 28), cirrhotic patients without hepatocellular carcinoma (*n* = 59), and those with hepatocellular carcinoma (*n* = 56). The cirrhotic patients (with or without hepatocellular carcinoma) were recruited from Medical Clinic 5 and from Surgical Clinic 1 of the Padua University Hospital. Cirrhosis severity, defined according to the well-known Child–Pugh score (CP), was classified into three stages: A (mild, score 5–6), B (medium, score 7–9), C (severe, score 10–15). One hundred and fifteen patients with cirrhosis were prospectively and consecutively enrolled, until a number of at about 20 patients in each group of Child–Pugh class (CP A/B/C) was reached.

Inclusion criteria were as follows: (a) cirrhosis (in presence or absence of HCC), diagnosed on the basis of the American Association for the Study of Liver Diseases (AASLD) radiological criteria [55] or histology, when appropriate at the moment of recruitment, without antiviral, chemotherapeutic, or surgical treatment; (b) non-active potus; (c) no infections, with the exception of viral infection by HBV/HCV; (d) no acute hepatitis or ongoing acute complications, such as encephalopathy or gastrointestinal bleeding; (e) no innate errors of metabolism; (f) absence of autoimmune diseases; (g) no oral hypoglycemic drugs; (h) no treatments with antibiotics, no immuno-suppressive drugs, no anti-inflammatory drugs or drugs that influence inflammation, and consequently, the levels of inflammatory cytokines; (i) no cardiovascular diseases (stroke, ischemic cardiopathy). Twenty-nine percent of enrolled patients had previously been diagnosed with type 2 diabetes mellitus.

Healthy controls (28 participants, all males, average age of 34 years, range 22–52 years old) were selected from hospital staff, on the basis of their clinical history, non-smokers, no alcohol abuse, no hypertension, and no diabetes, in absence of infections, and after the hematological tests. This study was approved by the Ethical Committee of Padua University Hospital with Protocol Number:1958P. Informed written consent was obtained from each patient and control, and the study was in accordance with the ethical guidelines of the 1975 Declaration of Helsinki.

Blood samples of the patients and controls were collected in EDTA tubes, after overnight fasting. All samples were immediately centrifuged (1500× *g*, 15 min, 4 °C), and the recovered plasma was frozen in liquid nitrogen and stored at −80 °C until further analysis.

### 4.2. Methods

#### 4.2.1. sICAM-1, sVCAM-1, TNF-α and IL-6 Quantification

sICAM-1, sVCAM-1, TNF-α and IL-6 quantification was performed using the bead-based multiplex immunoassay and the xMAP technology. Bio-Plex immunoassay kits, Hu Cyto Group I 2-plex (for IL-6 and TNF-α) and Hu Cyto Group II 2-plex (for ICAM-1 and VCAM-1) were from Bio-Rad (Milan, Italy). The assays were performed according to the manufacturer’s instructions by the Bio-Plex Service of C.R.I.B.I (Centro Ricerche Interdipartimentale Biotecnologie Innovative—Innovative Biotechnology Centre of the University of Padova), using the Luminex 100 system, for detecting fluorescence and the Bio-Plex Manager 6.2 Software for data analysis. The Bio-Plex human cytokine kits contained all reagents and standards required for the assay and for the specific calibration curves.

#### 4.2.2. sVAP-1 Protein Quantification and Amine Oxidase Activity Assays

sVAP-1 protein was quantified by an ELISA kit (VAP-1 Human Kit ELISA, Bender Medsystems, purchased from Prodotti Gianni, Milan, Italy). According to the manufacturer’s protocol, the samples were processed with the reagents, and the optical density was determined at 450 and 650 nm using a microplate reader.

sVAP-1/SSAO enzymatic activity was assessed using our unpublished fluorometric method, based on a modification of a previously proposed spectrophotometric assay [56]. In this assay, amine oxidase activity is expressed as generation rate of formaldehyde, the reaction product of VAP-1/SSAO activity, when methylamine is used as the specific substrate for human VAP-1. Formaldehyde is quantified by coupling the formaldehyde dehydrogenase/NAD^+^ enzymatic system and by detecting the correspondent NADH production by fluorimetry (details in Supporting Information). A Cary Eclipse fluorescence spectrophotometer (Varian Inc., Palo Alto, CA, USA) and a quartz cuvette with a 1 cm optical path were used for all the fluorescence measurements.

#### 4.2.3. Nitrite, Nitrate, and Oxidatively Modified Protein Determination

The nitrites and nitrates assay was performed using the commercial Nitrate/Nitrite Colorimetric Assay kit from Cayman Chemical (U.S.A.). The determination of carbonyl content in oxidatively modified proteins was assessed on the basis of its reaction with dinitrophenylhydrazine (DNPH), as previously described [57]. Firstly, 0.1 mL of plasma was added to 0.5 mL of 10 mM DNPH in 2 M HCl, then the solution was vortexed for one minute and kept at room temperature for 1 h. Then, 0.5 mL of 20% trichloroacetic acid (TCA) was added and the tube was centrifuged for 3 min at 10,000× *g*. The supernatant was then discarded, and the pellet was washed three times with 1.5 mL of ethanol–ethyl acetate (1:1) to remove the free reagent. After centrifugation, the precipitated proteins were dissolved in 0.5 mL of 6 M guanidine solution at 30 °C for 15 min. The insoluble material was removed by centrifugation and the absorbance of the solution was read at 370 nm (ε_370nm_ = 22,000 M^−1^cm^−1^, for the hydrazones formed by the reaction of DNPH with the protein carbonyls). The protein concentration in the solution was determined according to the method described by Bradford [58] using bovine serum albumin (BSA) as the calibration protein. The carbonyl concentrations were expressed in nmol _carbonyl_/mg of protein.

A Cary 50 Scan UV–visible spectrophotometer (Varian Inc., Palo Alto, CA, USA) was used for spectrophotometric measurements. All chemicals were analytical grade and were purchased from Sigma-Fluka-Aldrich (Milan, Italy).

#### 4.2.4. Statistical Analysis

Demographic variables and clinical features were analyzed using descriptive statistics. The χ^2^ test and Fisher exact test, as appropriate, were used to compare the categorical variables between two groups. The normality of distributions of continuous measures was tested using the Kolmogorov–Smirnov and Shapiro–Wilk test. Non-parametric tests were applied to compare each biomarker level between two (Mann–Whitney test) or more groups (Kruskal–Wallis nonparametric test, followed by Dunn’s multiple comparisons). Bivariate relationships among the biomarkers were examined using Spearman’s rho for continuous variables. A multiple linear regression model was used to identify predictors for the sVAP-1 protein (response variable). The explanatory variables introduced in the model were HCC diabetes (that expresses the combination of presence/absence of HCC and presence/absence of diabetes), TNF-α, IL-6, sICAM-1, and sVCAM-1. The results of the model are expressed as standardized Beta coefficients, the significance of the t-value associated with each, and the 95% confidence interval for unstandardized Beta coefficients.

All statistical tests were 2-sided, and a *p* value < 0.05 was used as the threshold for considering a difference as statistically significant. Statistical analyses were performed using IBM SPSS version 18.0 for Windows (SPSS Inc., Chicago, IL, USA).

## Figures and Tables

**Table 1:** Baseline demographics and clinical characteristics of healthy controls and of cirrhotic patients with or without hepatocellular carcinoma (HCC-1 and HCC-0, respectively).

Variable	Controls	(HCC-0)	(HCC-1)
Number (n)	28	59	56
Sex (male/female)	28/0	50/9	45/11
Age (y)	34 (22–52)	61 (41–80)	62 (35–89)
CHILD A/B/C ^1^ (n)	-	23/20/16	33/16/7
Diabetes (n)	0	11	21
ALT (U/L)	18 (10–35)	26 (7–71)	62 (14–482)
AST (U/L)	26 (10–35)	69 (18–566)	73 (14–482)
αFP (ng/mL)	n.d ^2^	2.6 (2.0–4.7)	710 (0.9–18372) ^3^

^1^ The Child–Pugh classification (CHILD) was used to classify the severity of liver disease in three sub-groups: A (compensated cirrhosis), B (significantly compromised liver function), and C (severe degree, significantly compromised liver function). Data are expressed as mean values and min–max range (in brackets); ^2^ not determined; ^3^ significantly different from cirrhotic without hepatocarcinoma group (HCC-0) (*p* < 0.05; *p* values were calculated by Mann–Whitney *U* test).

**Table 2:** Inflammatory cytokines and soluble adhesion molecules of non-diabetic cirrhotic patients with (HCC-1)’ or without hepatocellular carcinoma HCC (HCC-0)’.

Biomarker ^1^	(HCC-0)’ (*n* = 48)	(HCC-1)’ (*n* = 35)
IL-6 (pg/mL)	18 ± 20	14 ± 17
TNF-α (pg/mL)	3.2 ± 3.8	5.0 ± 1.6
sVCAM-1 (ng/mL)	433± 238	546 ± 266
sICAM-1 (ng/mL)	290± 161	300 ± 94
sVAP-1 (ng/mL)	762 ± 354	698 ± 320

^1^ From the comparison between the two cirrhotic groups *p* > 0.05 (Mann–Whitney test) was found for all variables.

**Table 3:** Correlation between the various biomarkers in patients with cirrhosis, grouped in relation to the presence of hepatocellular carcinoma (HCC-0 and HCC-1).

		sVCAM-1 (ng/mL)	IL-6 (pg/mL)	TNF-α (pg/mL)	sICAM-1 (ng/mL)
sVAP-1 (ng/mL)	All cases	0.293 **	0.089	0.040	0.193
	HCC-0	0.403 **	−0.030	0.020	0.298
	HCC-1	0.220	0.063	0.177	0.085
sVCAM-1 (ng/mL)	All cases		0.172	0.137	0.569 **
	HCC-0		−0.024	0.166	0.479 **
	HCC-1		0.533 **	0.047	0.716 **
IL-6 (pg/mL)	All cases			0.234 *	0.153
	HCC-0			0.485 **	0.075
	HCC-1			−0.141	0.304 *
TNF-α (pg/mL)	All cases				0.135
	HCC-0				0.169
	HCC-1				0.097

(*) *p* < 0.05; (**) *p* < 0.01. Bivariate relationships among the biomarkers were examined using Spearman’s rho. HCC-0 = patients without HCC; HCC-1 = patients with HCC.

**Table 4:** Linear relationships of sVAP-1 with the variables HCC, diabetes, adhesion molecules, and cytokines: multiple linear regression model.

	Standardized Coefficients (Beta)	*p*-Value	95% Confidence Interval
Constant		0.000	410.237–1013.815
HCC diabetes	−0.106	0.440	−114.799–50.575
IL 6 (pg/mL)	−0.065	0.631	−7.161–4.375
TNF-α (pg/mL)	0.014	0.914	−27.551–30.706
sICAM-1 (ng/mL)	−0.051	0.748	−1.095–0.791
sVCAM-1 (ng/mL)	0.374	0.021	0.073–0.877

In the model, sVAP-1 protein was the response variable. HCC diabetes, TNF-α, IL-6, sICAM-1, and sVCAM-1 were the explanatory variables. R^2^ = 0.110; adjusted R^2^ = 0.036.

## Data Availability

All data, if not already included in the manuscript, are available from the corresponding author on reasonable request.

## Supplementary Materials

The following supporting information can be downloaded at: https://www.mdpi.com/article/10.3390/ijms25137309/s1, Refs. [56,59] are cited in Supplementary Materials file.
