# Supplementary material for: Serum Vascular Adhesion Protein-1 and Endothelial Dysfunction in Hepatic Cirrhosis: Searching for New Prognostic Markers
Source: Int J Mol Sci. 2024 Jul 3;25(13):7309. doi: 10.3390/ijms25137309 (PMC11242677; doi:10.3390/ijms25137309)
Supplement: Supplementary file 1 [file ijms-25-07309-s001.zip › ijms-3025559-supplementary.pdf]

Serum Vascular adhesion protein-1 and **endothelial dysfunction in hepatic cirrhosis: searching for new prognostic markers**

Silvano Fasolato[1], Emanuela Bonaiuto[2,*] Monica Rossetto [2], Paola Vanzani[2], Fabio Ceccato[3] , Fabio Vittadello[4], Lucio Zennaro[2], Adelio Rigo[5], Enzo Mammano[3], Paolo Angeli[1] , Patrizia Pontisso[1], Maria Luisa Di Paolo[2,5]

Supporting information

S1. sVAP-1/SSAO activity assay method

The fluorometric assay to determine SSAO/VAP-1 activity in human plasma is based on a modification of a previously proposed spectrophotometric assay [1]. It was developed to determine SSAO activity in tissue extracts using methylamine (CH_3-NH_3^+), a specific substrate for human SSAO/VAP-1. The formaldehyde (HCHO) produced by SSAO activity (reaction 1) is oxidized to formic acid (HCOOH) by formaldehyde dehydrogenase (FDH) with the reduction of NAD^+, as follows:

1) $CH_3\text{-}NH_3^+ + H_2O + O_2 \xrightarrow{SSAO} HCHO + H_2O_2 + NH_3^+$

2) $\beta\text{–}NAD^+ + HCHO \xrightarrow{FDH} \beta\text{-}NADH + HCOOH$

The NADH generated by reaction (2) is continuously monitored at 340 nm (molar absorbance coefficient: 6220 M^{-1} cm^{-1}). We modified/adapted this method to determine SSAO activity in human plasma. Sensitivity was increased by fluorometric detection of NADH and the problems of low concentrations of SSAO/VAP-1 and of the intrinsic fluorescence of human plasma were overcome by setting up a discontinuous assay procedure. The standard protocol set-up is as follow: a plasma sample (300 μl) is pre-incubated for 20 min at 37°C with 0.5 mM pargyline, (inhibitor of monoamine oxidases) and the reaction was then initiated by the addition of 10 mM methylamine. This substrate concentration is the optimal to measure SSAO/VAP-1 in saturating condition for substrate (K_m(Methylamine) ≈ 0.7 mM [38]).

At intervals of 15 min, 30 μl of the sample are withdrawn from the plasma solution for the fluorimetric measurement of the amount of HCHO produced until that moment. At least three aliquots of plasma are sampled, for a total assay time of about 60 min.

The sampled aliquots are added to the assay solution containing 0.5 mM NAD^+ in 50 mM potassium phosphate, pH 7.20 (assay volume 830 μl) (all chemicals from Sigma-Fluka-Aldrich,

Milan, Italy). The fluorescence intensity of this solution is recorded as the baseline (Figure S1A).

After addition of formaldehyde dehydrogenase (FDH) from *Pseuodomonas putida,* the increase in fluorescence intensity caused by the quantitative oxidation of formaldehyde (HCHO) and correspondent production of NADH is recorded. From this increment of fluorescence intensity, the amount of HCHO produced by SSAO activity is calculated by a specific calibration curve obtained in the same plasma sample by adding standard HCHO (without methylamine). Figures S1B and inset C show an example of experiment carried out to obtain a calibration curve. Some examples of SSAO/VAP-1 activity determinations (HCHO generation rate) are shown in Figure S1D. We tested plasma at dilutions ranging from 10/890 to 60/890 (v/v). Calibration curves with mean slope values of (36.4 ± 6.2) $I_{f\,(a.u.)}/\mu M_{HCHO}$ (n =34 human plasma samples) were obtained for dilution 30/890. The same slope values were obtained when NADH or, alternatively, the freshly prepared formaldehyde, NAD^+ and FDH, were used.
To validate this method, SSAO/VAP-1 activity determinations were carried out in the presence and absence of SSAO/VAP-1 inhibitors, i.e. the "standard" semicarbazide (5mM) and the specific guanabenz (1mM) [2]. No HCHO were measured either in samples containing semicarbazide or in those containing guanabenz, confirming that the method reveals only the formaldehyde produced by SSAO/VAP-1 activity.
To test the applicability of this method to determine SSAO/VAP-1 activity levels in human plasma, we analyzed 27 heparinated samples (frozen at -80°C until analysis) from healthy volunteers and from patients with diseases associated with increased levels of circulating SSAO/VAP-1 (such as diabetes and inflammatory diseases, in addition to cirrhotic patients; all patients gave their informed and written consent). The ELISA assay showed good correlations between SSAO/VAP-1 activity and concentrations of circulating SSAO/VAP-1 protein (Figure S1E).
The fluorimetric method reported here was characterized by a linear response in time and by sensitivity of about 100 $pmoles_{HCHO}/(min \times ml_{plasma})$ (60 minutes assay time). A detection limit of about 0.2 μM of HCHO was calculated for the single measurement. This assay is well suited to detect SSAO/VAP-1 activity in human plasma: it is simple to use, does not require extraction procedures, avoids the use of radioactivity, and is also relatively inexpensive.

References:

1. Lizcano, J.M.; Unzeta, M.; Tipton, K.F. A spectrophotometric method for determination of the oxidative deamination of methylamine by the amine oxidases. *Anal Biochem* **2000,** *286*, 75-79. doi: 10.1006/abio.2000.4782.
2. Holt, A.; Smith, J.; Cendron, L.; Zanotti, G.; Rigo, A.; Di Paolo, M.L. Multiple binding sites for substrates and modulators of semicarbazide-sensitive amine oxidase: kinetic consequences. *Mol Pharm* **2008**, *73*:525-538.

Figure S1. Assay procedure to determine the amount of formaldehyde produced by SSAO activity in human plasma, with methylamine as substrate.
(A) Example of an assay: Baseline: fluorescence intensity of standard assay solution (NAD^+, 0.5 mM in potassium phosphate 50 mM, pH 7.2) containing 30 µL of a plasma sample (after 20 min incubation with 10 mM methylamine, at 37°C). After addition of formaldehyde dehydrogenase, HCHO produced in sample is oxidized to formate with an equivalent production of NADH and, consequently, increased fluorescence is measured. (instrumental set-up: λ_{ex}= 340 nm; λ_{em}=464 nm, excitation slit 10 nm, emission slit 20 nm).
(B) Example of experiment carried out to obtain calibration curves with human plasma samples. The baseline is the fluorescence intensity of assay solution containing plasma sample (30 µL) not incubated with substrate. After addition of FDH, various concentrations of freshly prepared HCHO were added, and corresponding increase in fluorescence intensity were recorded.
(Inset C) Calibration curve with standard HCHO: the increase in fluorescence intensity (B) are plotted *vs* formaldehyde concentrations. Calibration curve is result of linear regression through plotted data (in this example, intercept = 5 ΔI_f, slope= 40 $\Delta I_f/\mu M_{HCHO}$; r=0.9994) (Sigma Plot software, Version 10.0, Jandel Scientific, San Rafael, CA, USA). HCHO produced by SSAO activity in sample (shown in Figure S1A) is calculated from this type of calibration curve.
*(D). Application of method to measure SSAO/VAP-1 activity in some plasma samples from patients with various diseases***.** Concentrations of HCHO produced by SSAO activity, at various times, in plasma samples (incubated with substrate) were calculated from their calibration curves (see Figure S1). Straight lines: results of linear regression analysis through plotted data. Slopes of these lines: values of SSAO activity in plasma expressed in μM_{HCHO}/min (equivalent to $nmol_{HCHO}$/min· ml_{plasma}).
*(E). Correlation between SSAO/VAP-1 activity and concentrations of circulating SSAO/VAP-1 protein in human plasma from healthy individuals and patients with various diseases***.**
SSAO/VAP-1 activity in various samples was determined by fluorimetric assay; SSAO/VAP-1 protein concentration was determined by ELISA (commercial kit from Bender MedSystems GmbH, Vienna, Austria). Linear regression analysis shows good linear correlation between SSAO activity and SSAO/VAP-1 protein concentration (r=0.907, n=27).

Figure S1

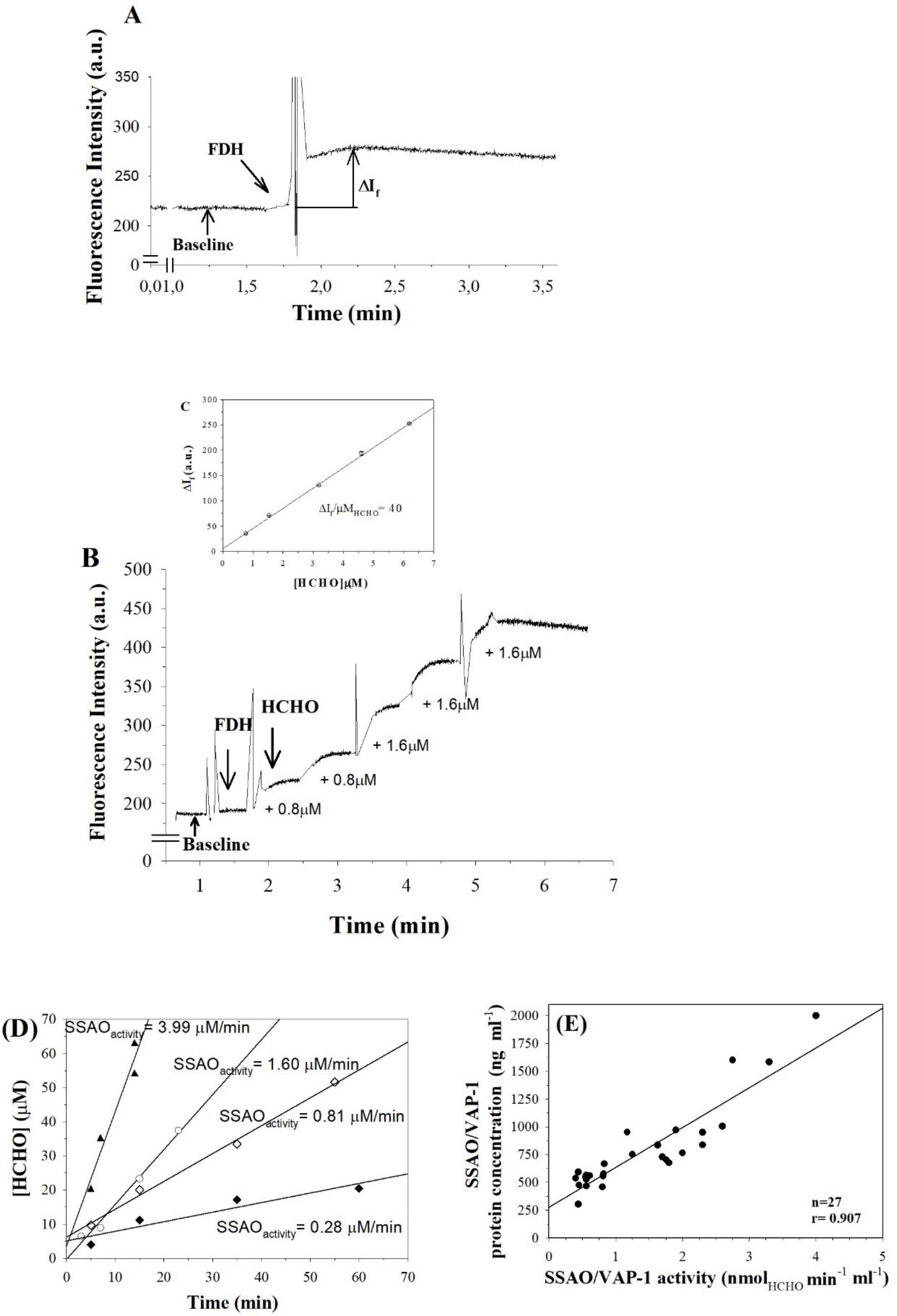
A
Fluorescence Intensity (a.u.)
Time (min)
FDH
Baseline
ΔI_f
0,01,0
1,5
2,0
2,5
3,0
3,5
C
ΔI_f (a.u.)
[HCHO] μM)
ΔI_f/μM_HCHO = 40
B
Fluorescence Intensity (a.u.)
Time (min)
FDH
HCHO
Baseline
+ 0.8μM
+ 0.8μM
+ 1.6μM
+ 1.6μM
+ 1.6μM
(D)
[HCHO] (μM)
Time (min)
SSAO_activity = 3.99 μM/min
SSAO_activity = 1.60 μM/min
SSAO_activity = 0.81 μM/min
SSAO_activity = 0.28 μM/min
(E)
SSAO/VAP-1 protein concentration (ng ml^-1)
SSAO/VAP-1 activity (nmol_HCHO min^-1 ml^-1)
n=27
r= 0.907

TABLE S1: **Serum concentrations of the biomarkers in cirrhotic patients with hepatocellular carcinoma with viral and non-viral etiology**. Data are expressed as mean values ± SD; p values were calculated by Mann-Whitney *U* test.

**Biomarker**	**Non Viral HCC-1 (n=31)**	**Viral HCC-1 (n=25)**	**P**
**IL-6** (pg/ml)	13±16	9±11	n.s.*
**TNF-α** (pg/ml)	5.6 ±2.9	7.2±5.8	n.s.
**sVCAM-1** (ng/ml)	665±281	602±320	n.s.
**sICAM-1** (ng/ml)	463± 484	371± 242	n.s.
**sVAP-1** (ng/ml)	754±288	782±406	n.s.

*n.s.=p>0.05

TABLE S2: **Serum concentrations of the biomarkers in cirrhotic patients with hepatocellular carcinoma with and without diabetes**

**Biomarker**	**Diabetes HCC-1 (n=31)**	**No-diabetes HCC-1 (n=25)**	**P**
**IL-6** (pg/ml)	13±16	9±11	n.s.*
**TNF-α** (pg/ml)	5.6 ±2.9	7.2±5.8	n.s.
**sVCAM-1** (ng/ml)	665±281	602±320	n.s.
**sICAM-1** (ng/ml)	463± 484	371± 242	n.s.
**sVAP-1** (ng/ml)	754±288	782±406	n.s.

*n.s.=p>0.05

TABLE S3: **Serum concentrations of the biomarkers in the three classes of severity, according to the Child-Pugh score, in cirrhotic patients with hepatocellular carcinoma (HCC-1) in absence of diabetes**
Data are expressed as mean values ± SD, p values from Kruskal-Wallis test.

Biomarker	CP A	CP B	CP C	p
**IL-6** (pg/ml)	5.6±6.5	4.4±4.3	27±26	n.s.*
**TNF-α** (pg/ml)	6.1±1.5	8.9±9.9	4.0±0.5	n.s.
**sVCAM-1** (ng/ml)	653±411	411±166	673±31	n.s.
**sICAM-1** (ng/ml)	456± 285	274±55	379± 102	n.s.
**sVAP-1** (ng/ml)	735±334	557±328	667±202	n.s.

*n.s.=p>0.05
